# Plant Metabolites and Regulation under Environmental Stress

**DOI:** 10.3390/plants10102013

**Published:** 2021-09-25

**Authors:** Ágnes Szepesi

**Affiliations:** Department of Plant Biology, Institute of Biology, Faculty of Science and Informatics, University of Szeged, Közép fasor 52., 6726 Szeged, Hungary; szepesia@bio.u-szeged.hu; Tel.: +36-62-544-307

## 1. Introduction

This Special Issue (SI) was planned to focus on abiotic stress-induced metabolic adjustments and their regulations in plants. The purpose was achieved by collecting and publishing 7 papers covering the recent progress and perspectives related to plant metabolites and their regulation during environmental stress conditions. This SI became very diverse: from molecular aspects to post-harvest applications, to beneficial applications of mycorrhizal fungus to study ibuprofen from human waste. This broad range of aspects and these current important topics could contribute to elucidate new strategies for developing climate-resilient crops to establish a sustainable agriculture under variable and unpredictable climate. This Editorial was written to emphasize the significance of articles published in this SI, stimulating further studies of plant metabolites and regulation during environmental stress conditions.

The relationship of plant and mycorrhizal fungus is very complex. Although beneficial effects of some ectomycorrhizal fungi have been described under abiotic stresses, the precise mechanism of this connection and related metabolites needs to be investigated. The work by Lorente et al. [1] provided evidence that ectomycorrhizal *Pisolithus tinctorius* could be effective to improve growth of *Cistus albidus*, a Mediterranean shrub, under water deficit. Their results suggest that the inoculation of *P. tinctorius* successfully improved the water status of plants. The value of scopoletin, a typical phytoalexin metabolite with antifungal activity, decreased in water stressed and inoculated plants showing induced defence mechanisms of these plants. Authors concluded that the use of *Cistus* plants inoculated with *Pisolithus tinctorius* could be a sustainable economic and environmental option for gardening or restoration projects.

Aromatic herbs provide high-value products with elevated contents of unique metabolites. Recent year studies of boosting levels of metabolites, such as flavonoids, alkaloids and glucosinolates of valuable herb plants, have increased by growing demand of human pharmaceutical or nutraceutical purposes [2]. *Polygonum minus* Huds. from Polygonaceae family, commonly known as kesum, is an aromatic herb plant from Southeast Asia. It is used as a spice and a flavouring agent to local cuisines, or as a traditional medicinal plants to treat digestive problems. Mohd Yusof et al. [3] have shown that *Polygonum* species are sensitive for shading, as biomass affected by low light positively altered the bioactive properties of this plant by 50% shading. Authors reported that shading was effective to increase the production of leaf pigments and secondary compounds, such as flavonoids contributing to the special adaptation to low light conditions. It can be concluded that micro-environmental manipulations, like different shading levels, could be a cost-effective and sustainable agricultural strategy to boost the bioactive compounds of valuable aromatic herb plants.

Human consumption and waste production could generate a novel threat to plants, the appearance of pharmaceutically active compounds in these extreme climate conditions. Christou and colleagues [4] elucidated that pharmaceutically active compounds could be act as stressors for plants inducing a new threat for agroecological systems. Ibuprofen (IBU) is one of the pharmaceutically active compounds, a nonsteroidal anti-inflammatory drug (NSAID) used excessively every year by the human population, contributing to elevated accumulation in the environment. Wijaya et al. [5] investigated the sensitivity of cowpea (*Vigna ungniculata*), one of the most important food legumes in semi-arid region of tropical areas, to IBU by ecotoxicological aspects. According to authors, *Vigna* developed several morphological and physicochemical adaptations to IBU stress in a dose-dependent manner, however, they concluded that the current range of IBU environmental pollution (0.5–6 ppm) could have a minimal effect on this plant. There are some questions that remained to be answered, e.g., IBU’s effect on plant anatomy, or its degradation in plants, or threat to food safety. This research could promote to know about new aspects of these pharmaceutically active compounds in other agriculturally important crops.

Post-harvest lignification process is detrimental for edible bamboo shoot, causing its quick deterioration, making it woody and inedible. Zhang et al. [6] compared the lignification process of two different bamboo species, high bamboo (*Phyllostachys prominens*) and moso bamboo (*Phyllostachys edulis*) in room temperature. Authors found that the lignification process was faster in high bamboo shoots than in moso bamboo, because of the increase of lignin and cellulose contents and shorter shelf-life. Furthermore, transcription network analysis revealed some genes which could contribute to the differences of lignification processes in these two bamboo species. This work stimulates further research to investigate the post-harvest mechanisms and methods for keeping optimal food quality of forest vegetable plants.

Crop plants play an important role in world food supply. Wheat and barley are the main crops which can feed millions of people and serve as animal feed. Zeeshan et al. [7] investigated the metabolic and transporter gene expression of high tolerance wheat and barley cultivars under salt stress, in order to discover the species/cultivar differences of salt stress responses. This contribution evidences the important role of glutathione homeostasis and TaWRKY10 transcription factor in salt tolerance of these species. These new findings could help us to develop salt tolerance in wheat and barley cultivars, enhancing the yield of these plants in salt affected regions in extreme climate conditions.

Protein synthesis and related posttranslational modifications are critical for proper function of proteins, helping plants to develop and grow, and also to survive during environmental stress conditions. Despite the great number of studies and data, there is a special modification which has relatively scarce information in plants. Hypusination is a unique posttranslational modification of eIF5A, a eukaryotic translation factor. During hypusination, hypusine, a rare amino acid, is synthesized by two enzymes, deoxyhypusine synthase (DHS) and deoxyhypusine hydroxylase (DOHH). Pálfi et al. [8] discussed the plant-specific details of hypusination and related signal pathways. Moreover, the diverse functions of eIF5A isoforms were overviewed in this review article, in order to emphasize the importance of hypusination and related functions in plants compared to animal studies. This review could be a promoter for further studies investigating the precise mechanism of hypusination in plants and its importance in plant stress responses. Furthermore, revealing some aspects of age-dependent or genotype-dependent alterations of hypusination could be useful for selecting stress-tolerant cultivars for agricultural production.

Current situations in the environment with climate change and harsher conditions could magnify the impact of abiotic stress factors to plants directly or indirectly affecting human population food production or food safety. Godoy et al. [9] summarized the applications of plant metabolites against abiotic stress factors, summarizing our current knowledge about potential strategies using plant metabolites, inducing tolerance of our crop plants. Applying these biostimulant plant metabolites, such as primary (proline, L-tryptophan, glutathione, citric acid) and secondary (polyols, ascorbic acid, lipoic acid, glycine betaine, α-tocopherol, melatonin), is sustainable and cost effective, however, more research and field experiment are needed to understand their precise mechanisms and related networks in plants. According to the authors, plant metabolites might provide a way to enhance these efforts to minimize the fertilizer supply and elevate the yield and food quality in a cost-effective and eco-friendly, sustainable manner. In this work, authors highlight the importance of different natural plant-derived compounds, expanding our knowledge about these valuable metabolites. This review further accelerates the current research of abiotic stress in crop species and efficient strategy of applying plant metabolites in the future, adding new aspects for agricultural and biotechnological value-added production.

## 2. Conclusions

In summary, these excellent articles could contribute to enhancing our knowledge about functions of plant metabolites in the future, to promote sustainable agriculture and improve food safety against extreme climate change. Moreover, this Special Issue could further promote studies of the biosynthesis and regulation of plant metabolites, as valuable sources of applications for green and sustainable agriculture and food production during diverse conditions under environmental stress.
